# Modelling the Impact of Antiretroviral Therapy on the Epidemic of HIV

**DOI:** 10.2174/157016211798038533

**Published:** 2011-09

**Authors:** Brian G Williams, Viviane Lima, Eleanor Gouws

**Affiliations:** 1 South African Centre for Epidemiological Modelling and Analysis (SACEMA), Stellenbosch, South Africa;; 2 British Columbia Centre for Excellence in HIV/AIDS and Centre for Health Evaluation and Outcome Sciences, St. Paul’s Hospital, Vancouver, British Columbia, Canada;; 3 Joint United Nations Programme on HIV/AIDS (UNAIDS), Geneva, Switzerland.

**Keywords:** ART, highly active antiretroviral therapy, HIV prevention, mathematical models.

## Abstract

Thirty years after HIV first appeared it has killed close to 30 million people but transmission continues unchecked. In 2009, an estimated 1.8 million lives were lost and 2.6 million more people were infected with HIV [1]. To cut transmission, many social, behavioural and biomedical interventions have been developed, tested and tried but have had little impact on the epidemic in most countries. One substantial success has been the development of combination antiretroviral therapy (ART) that reduces viral load and restores immune function. This raises the possibility of using ART not only to treat people but also to prevent new HIV infections. Here we consider the impact of ART on the transmission of HIV and show how it could help to control the epidemic.

Much needs to be known and understood concerning the impact of early treatment with ART on the prognosis for individual patients and on transmission. We review the current literature on factors associated with modelling treatment for prevention and illustrate the potential impact using existing models. We focus on generalized epidemics in sub-Saharan Africa, with an emphasis on South Africa, where transmission is mainly heterosexual and which account for an estimated 17% of all people living with HIV. We also make reference to epidemics among men who have sex with men and injection drug users where appropriate. We discuss ways in which using treatment as prevention can be taken forward knowing that this can only be the beginning of what must become an inclusive dialogue among all of those concerned to stop acquired immune deficiency syndrome (AIDS).

## INTRODUCTION

The development of antiretroviral drugs to treat Human Immunodeficiency Virus (HIV) has been a singular scientific achievement. Between 1995 and 2009 an estimated 14.4 million life-years have been gained globally among adults on antiretroviral therapy (ART) [2] but the rate of new infections is unacceptably high and still exceeds the number of people starting ART each year [3].

Many interventions to reduce transmission have been tried and tested over the last three decades [4]. The impact of treating sexually transmitted infections on HIV incidence remains equivocal [4]; behaviour change programmes have shown a demonstrable impact on HIV incidence in only a few countries [5]; medical male circumcision is effective, is being made available in much of Africa [6], and reduces the risk of HIV infection in men by 60% [7]; a vaginal microbicide containing tenofovir gel reduces the risk of HIV infection in women by 54% [8] while pre-exposure prophylaxis gives a similar reduction in men who have sex with men [9]; condoms are effective, if used correctly and consistently, and some interventions with sex workers have resulted in high levels of condom use [10], but the use of condoms in primary partnerships remains low [11]; counselling and testing is effective in reducing risk behaviour among HIV-positive but not among HIV-negative people [12]; antiretroviral treatment has had little impact on sexual transmission because it is currently provided in the late stages of infection [13]; an effective vaccine may still be decades away in spite of recent progress [14]. None of these interventions on their own are sufficient to eliminate HIV transmission although in combination there will be important synergies. One intervention that has succeeded is the use of ART to prevent mother-to-child transmission (MTCT) and in developed countries less than 2% of children born to HIV-positive mothers are themselves infected [15].

In this paper our focus is on the use of ART to suppress the viral load in infected people and to reduce the likelihood that they will infect their sexual partners or their children. We consider the magnitude of the control problem, i.e. the extent to which the risk of infection needs to be reduced in order to eliminate transmission. We outline dynamical models that have been developed to project the impact of ART on the epidemic, and examine the data that determine the parameters to be used in transmission models. Finally, we discuss the evidence that early treatment with ART reduces population level incidence. The analytical discussion is kept simple to illustrate general principles and reach general conclusions. Details of particular models can be found in the literature referred to here.

## THE MAGNITUDE OF THE CONTROL PROBLEM

The basic case reproduction number, *R*_0_, is the expected number of secondary cases if one primary case of infection is introduced into a fully susceptible population: if *R*_0_ can be reduced to, and then kept below, 1 an established epidemic will decline exponentially to zero; if *R*_0_ is greater than 1 a new epidemic will increase exponentially until it reaches saturation at a level determined mainly by *R*_0_ and the extent of heterogeneity in transmission between people [16]. To estimate *R*_0_ we note that if the initial epidemic doubling time is *D*, then each person infects, on average, one other person in a time *D*/ln(2); the factor of ln(2) depends on the assumption that prevalence increases exponentially at the start of the epidemic. If the life expectancy of people who are infected with HIV is *L*, then each person will infect, on average, *R*_0_ = ln(2)*L*/*D *people before they die [17]. Since some of those infected will die during the period *L* a better approximation [16] is

1 $$R_{0}=\frac{\ln\left(2\right)L+D}{D}$$

For people infected between the ages of 20 and 30 years, who do not receive ART, *L* is approximately 10 years irrespective of gender, mode of transmission or country [18]. For heterosexual epidemics in nine African countries the initial doubling time is *D* ≈ 1.0 year [19] giving *R*_0_ ≈ 8. *R*_0_ may be higher in epidemics of HIV among men who have sex with men and a recent study in Norway suggests a value of 15 [20]. To eliminate a heterosexual epidemic we therefore need to reduce transmission by about 10 times, or 90%, and maintain this reduction until all those who are being kept alive through the use of ART have died, one hopes of natural causes.

## METHODS OF CONTROL

### Sexual Transmission

There have been dramatic declines in HIV prevalence and incidence in heterosexual epidemics in some countries, most notably in Zimbabwe [21]. The reasons for this are not clearly understood but there is evidence of concomitant changes in sexual behaviour [22]. In countries where risky sexual behaviour has declined among young people there is evidence of a corresponding decline in the incidence of infection [23] but further research is needed to understand these changes. People who know their HIV status are less likely to engage in risky sexual behaviour, especially with their regular partners [24,25], but national population based surveys in sub-Saharan Africa show that less than 40% of people living with HIV know their status [26].

Widespread, consistent and proper condom use would dramatically change the course of the epidemic, and probably contributed to the control of HIV in Thailand [10], but evidence for population-level impact in other places is weak [11]. There is evidence that treating curable sexually transmitted infections can reduce the incidence of HIV [27] but not by much. Male circumcision reduces transmission from women to men by 60% (95% confidence interval [CI] 46%–70%), corresponding to an overall reduction of 37% (95% CI 27%–45%) [7,28,29] which is significant but not sufficient to eliminate HIV transmission [17]. A tenofovir-based vaginal microbicide reduced transmission from men to women by 54% (95% CI 4%–80%) corresponding to an overall reduction of 32% (95% CI 2% to 67%) among those with greater than 80% compliance [8,30]. Emtricitabine and tenofovir taken orally, reduced sexual transmission between men by 44% (95% CI 16%–63%) [9] but a trial of oral pre-exposure prophylaxis for women in East Africa was stopped early because the interim analysis showed no evidence of effect [31]. In spite of the disappointing results from East Africa, pre-exposure prophylaxis could play an important role in preventing infections provided high levels of adherence can be achieved. The very high rates of HIV in southern Africa, which includes the nine worst affected countries in the world, may be due in large part to the migrant labour system in this region which separates men from their families for much of their adult lives [32] making it more likely that they will visit sex workers and that their wives will sell sex to buy food and support their children [33]. Dealing with the social disruption and psychological trauma associated with the system of oscillating migration in southern Africa, originating in the need for labour to serve the gold mines, could help to reduce sexual transmission in that region [34].

### Mother-to-Child Transmission (MTCT)

Without interventions, children born to HIV-positive mothers have a 25% to 45% chance of being infected during birth or while the mother is breastfeeding [35-37]. This can be reduced to less than 2% through elective Caesarean section delivery [15,38], by avoiding breastfeeding [39] and with provision of antiretroviral therapy to the mother or prophylaxis to the child [39]. However, Caesarean sections are not routinely available or safe in most of the worst affected countries of the world, and formula feeding is often neither safe nor feasible in resource-limited countries and can increase the risk of malnutrition or mortality from other infectious diseases [40,41]. Antiretroviral drugs given to the mother or the child can reduce transmission significantly [35,36] and the World Health Organization (WHO) now recommends that all HIV-positive mothers should be provided with either mono-ART (zidovudine) during pregnancy followed by nevirapine and lamivudine during and immediately after labour, or triple ART to reduce HIV transmission, starting as early as the second trimester of pregnancy [42]. In the studies on which these recommendations were based ART was started at or during the third trimester of pregnancy. Two factors favour starting ART as soon as possible during the course of the pregnancy. First, the viral load drops by 100 times during the first month of therapy but a further 100 times during the next eight months [43]. Second, after starting ART a person’s immune system only recovers slowly, with CD4 cell counts typically increasing by 100 cells/mm^3^ over the first year but continuing to increase over the next six or seven years [44]. We note also that since intermittent treatment is detrimental [45] once started on ART, it has been suggested that pregnant women infected with HIV should be kept on ART for life [46].

Of children born with HIV, about half will die before the age of 2 years and about half will die at a median age of 16 years if they are not given ART [47]. Starting all HIV-positive children on ART, as currently recommended by the WHO, must be a high priority. A model of MTCT transmission was used to predict the age specific prevalence of HIV in three countries in southern Africa, confirming that this was the main reason for the high rates of infection in children and adolescents [47].

### Injection Drug Users (IDU)

The risk of HIV among IDU can be reduced with treatment for drug abuse, HIV testing and counselling, peer outreach and ensuring access to sterile injection equipment [48]. Access to ART appears to be disproportionately low among IDUs because of systemic and structural obstacles, and IDUs infected with HIV often have an increased risk of death even in countries with good ART delivery systems [49]. A study of haemophiliacs infected through blood transfusions showed that transmission was 85% lower in people with a viral load below 10^5 ^copies/mL than in those with a viral load above 10^5^ copies/mL [50] and it is likely that ART will also reduce HIV transmission in people infected through the use of contaminated needles.

## TREATMENT AS PREVENTION (TasP)

In 1995, Ho called for the early treatment of HIV-positive people with antiretroviral drugs [51] on the grounds that it would limit the development and spread of drug resistance and lower the viral load set point while prolonging the life of the infected person. Ho was concerned primarily with the individual prognosis rather than with the impact on transmission. At that time the antiretroviral drugs reduced viral load by 10 to 100 times while the drugs can now reduce viral load by 10,000 times or more [43] so that the expected impact on transmission will be correspondingly greater.

By 2002 it was clear that ART reduces both viral load and transmission but the magnitude of the reduction was not clear, the prospect of creating drug resistance was of concern, and the scale of the task was daunting [52,53]. Some authors considered the impact of ART assuming that it would reduce transmission by anywhere between 1% and 99%, and showed that ART could substantially reduce and possibly eradicate HIV, even with high levels of drug resistance and risky sex [54,55], but a great deal depends on whether the reduction is 1% or 99%.

In 2006 Montaner *et al. *[56] published the first paper in which the use of ART to control and manage the epidemic of HIV was considered in detail. They argued that given the efficacy of new drugs, if all HIV-infected people were given ART within one year of seroconversion, the number of HIV-infected people could be reduced from 38 million to less than 1 million by 2050 and the cost would fall from an initial high of $15 billion *per annum* to $1 billion *per annum* with a total cost up to 2050 of $338 billion. Acknowledging the magnitude of the task, they argued that this approach merits consideration if it can offer a means to control the pandemic. Cohen *et al. *[57] were more cautious but agreed that the use of ART would evolve as a cornerstone of HIV prevention.

Blower *et al. *[58,59] were concerned that widespread use of ART could lead to the development of drug resistance, as is inevitable when infectious diseases are treated with drugs, and predicted that the clinical burden of ART resistance in San Francisco could reach 42% in 2005 [58]. A more recent modelling paper by the same group suggested that currently circulating non-nucleoside reverse transcriptase inhibitor (NNRTI)-resistant strains in San Francisco could pose a great and immediate threat to global public health but did not present any direct comparison of the model predictions with empirical trend data [60]. Fortunately, the prevalence of primary drug resistance in San Francisco only reached 13% (95% CI 8% to 31%) in 2006 [61]. Data from an ongoing, observational cohort study of HIV-infected patients receiving care in participating HIV clinics in eight United States (US) cities show that starting ART at CD4 counts above 350 cells/mm^3^ can substantially reduce the likelihood that a person will acquire drug resistance as a result of treatment [62] so that early treatment may limit, rather than enhance, the spread of resistance to HIV drugs.

While the widespread and early use of antiretroviral drugs could potentially eliminate sexually transmitted HIV, few data are available to demonstrate and directly measure the population-level impact of treatment on the incidence of HIV. We therefore rely on mathematical models to inform the design of suitable trials to explore the potential of this approach, to evaluate impact, to help in the design of field studies, and eventually to formulate policy. Models can also be used to highlight potential weaknesses in the use of treatment as prevention which may compromise its impact.

In public health it is important that the interests of the patient are not sacrificed to the general interest, and any discussion of using ART for prevention must also consider the extent to which early ART improves or compromises the prognosis for the individual patient.

## MODELLING THE IMPACT OF ART ON TRANSMISSION

### Heterosexual Transmission

ART can be used both to render those already infected with HIV less infectious (Treatment as Prevention or TasP) and those not yet infected with HIV less susceptible to being infected (Pre-exposure prophylaxis or PrEP).

#### Treatment as Prevention

To investigate the potential and the pitfalls of using ART for prevention, scientists at the WHO developed a dynamical model of transmission that they fitted to trend data for South Africa while drawing on parameter estimates from a wide range of studies [63]. South Africa was chosen because it has the greatest number of HIV-positive people in the world and has good trend data from the annual antenatal clinic surveys started by Küstner *et al. *in 1990 [64,65]. The WHO study [63] confirmed the earlier conclusions reached by Montaner *et al. *[56] and showed that annual HIV testing and immediate treatment could reduce HIV incidence and mortality in South Africa to less than 1 case per thousand people per year in five years and HIV prevalence to less than 1% in forty years. The estimated cost for South Africa, which accounts for 17% of global infections, was US$72 billion, 20% of the previous global cost estimate [56].

The WHO model [63] was also used to identify factors to which the result is most sensitive and the extent to which they might compromise the strategy (supporting information in [63], available on request). The model [63] was fitted to time trends in the prevalence of HIV among adults in South Africa as reported by the Joint United Nations Programme on HIV/AIDS (UNAIDS) [66]. For comparison we assume that in addition to the roll-out of ART there will be a 40% reduction in transmission over the next ten years, arising from expanding access to male circumcision [67], use of microbicides by women, and behaviour change resulting from awareness of HIV. This baseline scenario is shown in Fig. (**1**). The model then provides estimates of incidence and mortality and allows for projections into the future. Fig. (**2**) shows how the impact of ART on the epidemic varies if people start ART when their CD4 cell count is below 200 /mm^3^, as in the 2006 WHO treatment guidelines [68], 350/mm^3^, as in the 2009 WHO treatment guidelines [69], 500/mm^3^, or as soon as they test HIV positive. With annual testing and universal access, defined as 80% coverage of the population in need [3], starting treatment at 200 CD4 cells/mm^3^ should reduce the incidence in 2050 by 39%, the prevalence of HIV-positive people not on ART by 63% and the mortality by 83%. The impact on mortality is greater than on prevalence and incidence because most people will start treatment early enough to keep them alive but not early enough to substantially reduce transmission. Starting treatment at 350/mm^3^, the corresponding declines in incidence, prevalence and mortality by 2050 are 59%, 85% and 94%; starting at 500/mm^3^ they are 85%, 95% and 98.8%; and starting immediately they are 99.3%, 99.4% and 99.8%. In order to save lives, starting people on ART at 350/mm^3^ will provide most of the benefit; in order to stop transmission people should start ART as soon as they are found to be HIV-positive.

The sensitivity analysis in the WHO paper [63] showed that the most critical factors for controlling the epidemic were the reduction in transmission, the drop-out rate and the HIV testing interval. If we want to ensure that the steady state incidence is reduced by at least 90%, we need to ensure that the drop-out rate is less than 5.3% per year, the reduction in transmission is at least 95%, and the testing interval is less than 2 years, other things being equal. The results are less sensitive to the number of people that refuse treatment since mortality among this group will be high.

The results of the WHO model [63] were broadly confirmed [70] with a model showing that the impact of treatment depends on the extent of heterogeneity in sexual behaviour, which in turn reflects the structure of the sexual network; if there is little heterogeneity the impact of the intervention may be greater, and if there is much heterogeneity the impact may be less. Where sexual behaviour is very heterogeneous, targeting those at greatest risk of being infected or of infecting others will increase the impact of ART on transmission. Confirmation of the overall result was also provided by an age-structured model, fitted to data from South Africa [71], showing that it might be possible to achieve the same result with less frequent testing and raising the possibility of increasing the impact by targeting people in age groups at greatest risk of infecting others.

A model to forecast the impact of early treatment on HIV in Washington, District of Columbia (DC) [72] gave a more pessimistic outcome and suggested that regular testing and immediate treatment would be insufficient to eliminate HIV. However, the authors assumed that people are either fully infectious or uninfectious and that ART reduces the time spent with transmissible virus by only 65%, giving results similar to the reduction predicted for South Africa if treatment is started only when people’s CD4 cell count is below 350/mm^3^.

#### Treatment as Prophylaxis

Two trials of pre-exposure prophylaxis have given positive results. The *Centre for the AIDS Programme of Research in South Africa* (*CAPRISA) 004* trial showed that tenofovir gel, used as a vaginal microbicide, provided a significant level of protection to women against HIV-1 [8] and *Herpes simplex* virus (HSV)-2 [73] infection, offering a female-controlled method for reducing the risk of both viruses. It has been argued that the use of microbicides by women could benefit men more than women [74], but this result depends on the assumption that microbicides also reduce the chance that women will infect their male partners and that drug resistant HIV is less transmissible than drug susceptible HIV [75]. Here we only consider the impact that the gel might have as a consequence of the reduction in male-to-female transmission. Among women in the trial who used the gel in 80% or more of sexual contacts the incidence was reduced by 54% (95% CI 4%–80%), and in those who used the gel in 50% of sexual contacts the reduction was roughly halved. The reduction of 54% in one direction only (men to women) corresponds to a reduction of 32% (95% CI 2%–67%) averaged in both directions [16,17,30]. Using a model previously developed to study the impact of male circumcision [17], we can show that with full coverage and high adherence, the use of the gel would reduce the long term, steady-state prevalence, incidence and mortality of HIV infection by 30% to 35% over the next 20 years [30]. Over twenty years the use of tenofovir gel in South Africa could avert up to 2 million new infections and 1 million AIDS deaths, an important and significant contribution to the control of HIV but not enough to eliminate transmission when used in isolation from other prevention methods.

More recently [9] the *Preexposure Prophylaxis Initiative (iPrEx)* trial has shown that FTC and TDF combination therapy given to the HIV-negative partner in a discordant male couple can reduce the risk of HIV by 44% (95% CI 16% to 63%). In epidemics of HIV among men who have sex with men this can protect all sexually active people in the network so that the impact should be somewhat greater than that of the microbicide gel. For both forms of pre-exposure prophylaxis, further trials are needed in order to increase the impact, possibly by finding better ways to deliver the antiretroviral agent but also to get more precise estimates of the actual impact.

**Fig. (2):**
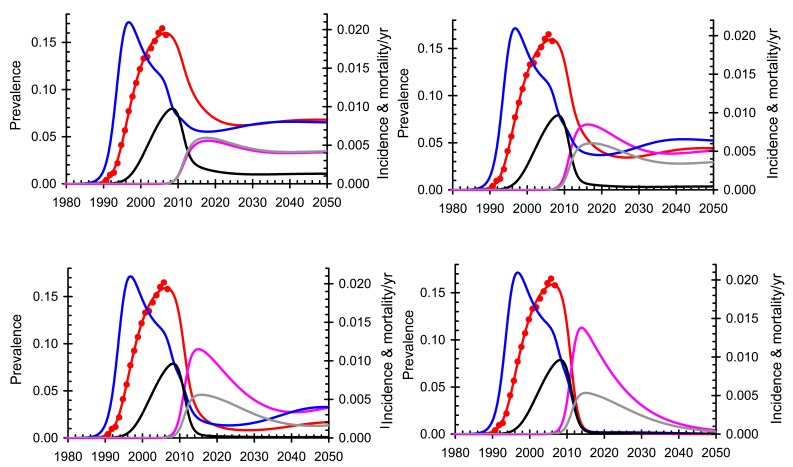
Impact of treatment on HIV in South Africa. People start antiretroviral therapy (ART) when their CD4 cell count is below 200 cells/mm^3^ (top left), 350 cells/mm^3^ (top right), 500/ cells/mm^3^ (bottom left) or if they start immediately, irrespective of CD4 cell count (bottom right). HIV prevalence not on ART (red), data (red dots) and on ART (pink); HIV incidence (blue); AIDS-related mortality not on ART (black) and on ART (grey).

**Fig. (3):**
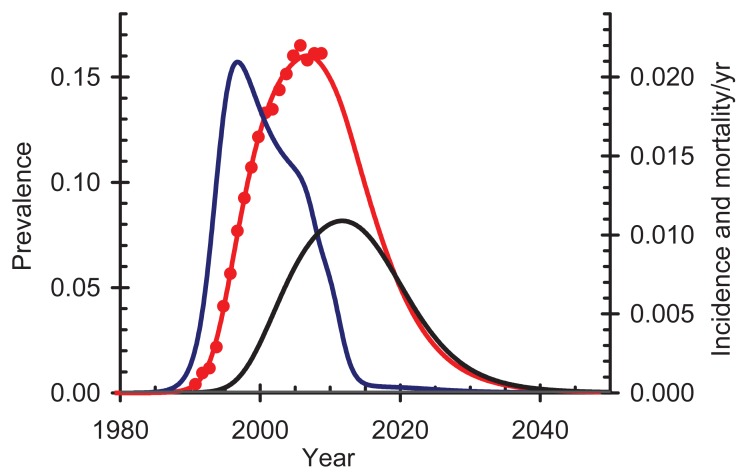
Impact of pre-exposure prophylaxis (PREP) on HIV in South Africa. All HIV-negative people are put onto antiretroviral therapy (ART). HIV prevalence (red); data (red dots); HIV incidence (blue); AIDS-related mortality (black).

In order to compare treatment as prevention (TasP) and treatment as pre-exposure prophylaxis (PrEP), we use the same model as before [63], with the same parameter values, and assume that a form of PrEP is available that gives 99% protection against infection. While the level of protection in this thought experiment is optimistic [9], we use it only for the purpose of comparison. Comparing PrEP (Fig. **3**) with immediate treatment (Fig. **2**; bottom right) shows that, as expected, the impact on incidence is almost the same with both interventions. However, with PrEP alone total AIDS related mortality is much higher than with treatment since those already infected will die, at least in this thought experiment. Implementing and delivering PrEP will be challenging, especially where the prevalence of infection is low, since many HIV-negative people would have to be given PrEP to stop relatively few infections. It also raises concerns about wide-scale HIV testing, safety screening, adherence and risk behaviour [76]. In practice it would be unethical to test many people and provide drugs to those who were HIV-negative but not to those who were HIV-positive. The analytical problem is to determine the optimal combination of these strategies; PrEP might be favoured when prevalence is low and incidence is high, for example among young girls in southern Africa, while treatment would be favoured when prevalence is high and incidence is low, for example among older men, but the best strategy needs further investigation.

### Mother-to-Child Transmission (MTCT)

**Fig. (4):**
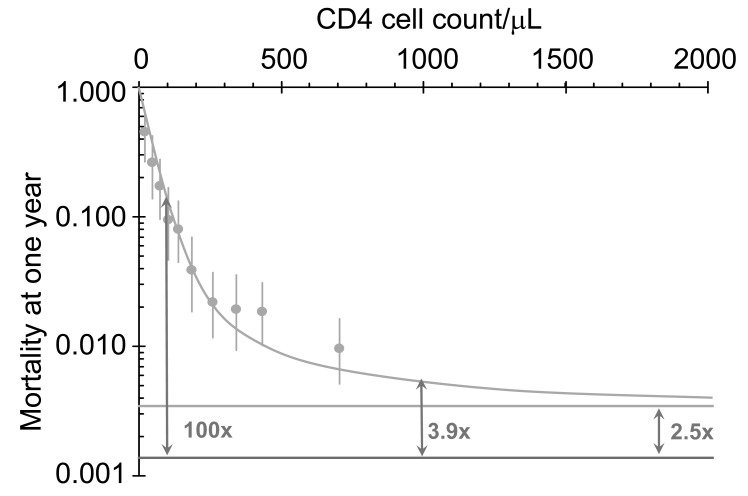
Survival of postpartum mothers in Harare, Zimbabwe (1997 to 2000) as a function of CD4 cell counts without treatment [80,81]. Dots: HIV-positive data; Fitted line: model; Upper horizontal line: asymptote; Lower horizontal line: HIV-negative mortality from data.

Without appropriate interventions, HIV kills mothers and infects their babies [77]. Here we estimate the impact of early treatment on maternal mortality and MTCT in South Africa. With a population of 49 million adults and an annual birth rate of 2.0% per person, there are about 1.0 million births per year [78]. The prevalence of HIV among women attending public antenatal clinics is 29% [79] so that approximately 250 thousand HIV-positive women give birth each year. Using data on mortality as a function of CD4 cell counts in pregnant women from Zimbabwe [80,81] (Fig. **4**), we estimate the number of women who will die within one year of giving birth without ART as a function of their CD4 cell counts, assuming conservatively that ART halves mortality.

Using data on the probability of MTCT six weeks after birth as a function of CD4 cell counts in pregnant women from various studies in Africa [35,36], we also estimate the number of women who will infect their children, with or without ART, as a function of their CD4 cell counts. Estimates of mother’s mortality and the proportion of infants that are infected with HIV in South Africa are shown in Fig. (**5**). Without ART, an estimated 6.8 thousand mothers will die each year within one year of giving birth. If all those whose CD4 cell count is below 350/mm^3^ are given ART the number of deaths will be reduced to 4.0 thousand and if all HIV-infected mothers are given ART this will be reduced to 3.4 thousand. Because mortality increases rapidly as CD4 cell counts decline, the biggest reduction will be obtained by ensuring that women with low CD4 cell counts are started on ART. Without ART an estimated 58 thousand children will be HIV-positive at six weeks of age each year. If all mothers with a CD4 cell count below 350/mm^3^ are started on ART before the birth of their child, transmission will be reduced to an estimated 33 thousand per year, and if all mothers are given ART regardless of CD4 cell count this will be reduced to 10 thousand per year. If HIV-positive mothers breastfeed their babies this will further increase the chances that their babies will be infected with HIV, but in some settings will reduce mortality from malnutrition, diarrhoea and pneumonia [40].

### Men who have Sex with Men (MSM)

A model of the impact of ART on the epidemic of HIV in the United Kingdom where there is transmission among men as well as between men and women [82] matched the observed 50% to 60% decline in deaths and new infections after 1995 as well as the more recent slight increase in new infections. Since people have been starting ART only at low CD4 cell counts, this model could be used to investigate the effect of earlier treatment. A recent model of HIV in MSM in Australia [83] shows that while the epidemiological impact of increasing treatment in the acute phase of HIV may be modest, increases in HIV testing rates could have substantial epidemiological benefits making this a highly effective public health intervention. A model of the MSM epidemic in the Netherlands suggested that the epidemiological benefits of highly active ART and earlier diagnosis on incidence have been offset by increases in risk behaviour rate [84]. However, it appears that the mean time to diagnosis was about three years and 90% of new infections were from 24% of men who do not know their HIV status, so that more frequent testing and better treatment coverage could have a substantial impact on this epidemic. Finally, a stochastic simulation model using trend data on viral load among MSM receiving treatment in the Netherlands showed that despite a significant reduction in transmission, the total lifetime risk of infection is still 22% (95% CI 9%–37%) if men do not use condoms and can be reduced to 3% (95% CI 0.2%–8%) with consistent condom use [85].

### Injection Drug Users (IDU)

A model of the impact of treatment on transmission in British Columbia, where the epidemic is concentrated predominantly among MSM and contacts of IDUs, was used to explore the implications of the International AIDS Society (IAS)-USA guidelines under which people would start ART if their CD4 cell count is less than 350/mm^3^, CD4 count has fallen by more that 100/mm^3^ in a year, or if their viral load is greater than 10^5^ copies/mL [86]. The model shows that with 75% ART coverage, the incidence of HIV would be reduced by about 85% within five years and would remain at this much lower level thereafter.

### Impact on Tuberculosis

Tuberculosis (TB) is the most common opportunistic infection associated with HIV [87] and the model developed for HIV [63] was extended to include TB [19]. The epidemic of HIV drives the epidemic of TB. The key assumptions regarding TB are that the incidence of TB increases by 36% for each drop of 100 CD4 cells/mm^3 ^as infection with HIV progresses, and that ART reduces the incidence of TB by 61% (95% CI 54%–67%) [19]. Fig. (**6**) shows the impact on TB in South Africa if people are tested annually for HIV and start ART when their CD4 cell count is below 200, 350 or 500/mm^3^ or if they start treatment as soon as they are found to be HIV-positive. Since the median CD4 cell count at which HIV-positive people present with TB is about 200/mm^3^ [88], most of the short term reduction in AIDS-related TB is gained if people start ART when their CD4 cell count is below 350/mm^3^, but eliminating AIDS-related TB in the long term will rely on eliminating HIV.

## PARAMETER ESTIMATION

Conclusions drawn from models depend on the reliability of the parameters used in the models. Here we discuss what is known about the key parameters that determine the impact of ART on HIV transmission.

### Adherence

Without high levels of adherence, early treatment will not reduce transmission [89]. By prolonging life without suppressing viral load, low levels of adherence could increase transmission and amplify drug resistance. However, several studies have shown that high levels of compliance can be reached. In British Columbia, 57% of people on NNRTI regimens and 69% on protease inhibitor regimens were more than 95% adherent [90]; in Mozambique 72% of patients were more than 95% compliant, based on pill counts [91]; while in Zambia 84% of patients were more than 95% adherent in their first months of ART. A review of 58 studies showed that mean levels of ‘adequate’ adherence, generally taken to be greater than 95%, were better in Africa than in North America (77% versus 55%, respectively) [92]. However, in British Columbia, being less than 95% adherent increased two-year mortality by 3.1 (95% CI 2.0 to 5.1) times. Even where adherence is high, ways should be found to increase this further [93] for the benefit of individuals, to minimise transmission and to limit the spread of drug resistance.

### Risk Compensation

Some researchers have expressed concern that risk behaviour may increase when people start ART, and increases in risk behaviour have been observed among MSM in developed countries [84,94,95]. However, a review of 25 studies including heterosexual men and women, MSM and IDU showed no overall increase in sexual risk behaviour among those receiving ART [96]. There was no difference in rates of unprotected sex among HIV-infected people receiving or not receiving ART (odds ratio [OR] 0.92, 95% CI 0.65–1.31) or between those with an undetectable viral load compared to those with a detectable viral load (OR 0.99, 95% CI 0.82–1.21). Some studies have shown that ART has been associated with a decrease in sexual risk behaviour. In Uganda, starting people on ART reduced the likelihood that they would engage in risky sexual behaviour by 50% to 70% [24,25]. A seven-year study in South Africa found that after ART initiation, people had less unprotected sex (OR 0.4, 95% CI 0.34–0.46) and fewer multiple sexual partners (OR 0.2, 95% CI 0.14–0.29) [97]. Expanding ART provision could therefore enhance HIV prevention in some settings. It is, nevertheless, essential that people who start ART receive proper risk-reduction counselling and support to ensure that the gains from ART are not lost to increases in risk behaviour.

### Viral Load Suppression

The impact of treatment on prevention depends on the extent to which ART suppresses a person’s viral load, making that person less infectious to others, and with currently available drugs there is evidence that high levels of viral load suppression can be achieved [43,98]. Results from viral load measurements are more widely available in blood plasma than in semen or vaginal secretions, but the plasma viral load is strongly correlated with the other two [99,100]. If HIV-positive people take antiretroviral drugs and are fully compliant, it is possible to reduce their viral load by four to five orders of magnitude and to maintain it at these low levels for several years [43]. In the US military, the median viral load before ART initiation was 25,000 copies/mL and after eight years on ART, 82% of 735 patients had undetectable viral load (< 400 copies/mL) [98]. In 89 studies of adult patients in ART programmes in sub-Saharan Africa, with a follow up of 6 months to 2 years, the median proportions of patients with viral loads below 1000 copies/mL, 400 copies/mL and 50 copies/mL were 85%, 79% and 69%, respectively [101].

When HIV-positive people are co-infected with other pathogens, their HIV viral load increases. Treating co-infections reduced the viral load in people with TB, malaria, geohelminths, schistosomiasis, filariasis, *Herpes simplex* virus, gonorrhoea, and syphilis [102,103]. However, the median reduction was only 2.7 times (interquartile range [IQR] 1.8–5.1) which will make a small but significant contribution to viral load reduction—the one exception was a study of TB treatment that gave a reduction of 3000 times [102]. Co-infections should always be treated but it seems unlikely that this will reduce HIV transmission significantly.

### Residual Transmission

#### Heterosexual

There is evidence that transmission levels off at viral loads above about 10^5^ copies/mL [104]. Fig. (**7**) shows data from three recent studies, one being a meta-analysis [103,105-107]. A biologically plausible model that fits the observed data assumes that the probability of transmission is proportional to the viral load when the viral load is low, but converges exponentially to an asymptote at high viral loads. The relationship between transmission, *T*, and viral load *V*, is then

2 $$T=\alpha \left(1-{\mathrm{e}}^{-\beta V}\right)$$

where *α*is the asymptotic value of the transmission rate and transmission is proportional to *αβV* at low viral loads. The fits to the data are shown in Fig. (**7**). Because transmission saturates at high viral loads, the reduction in transmission will be less than the reduction in viral load.

**Fig. (7):**
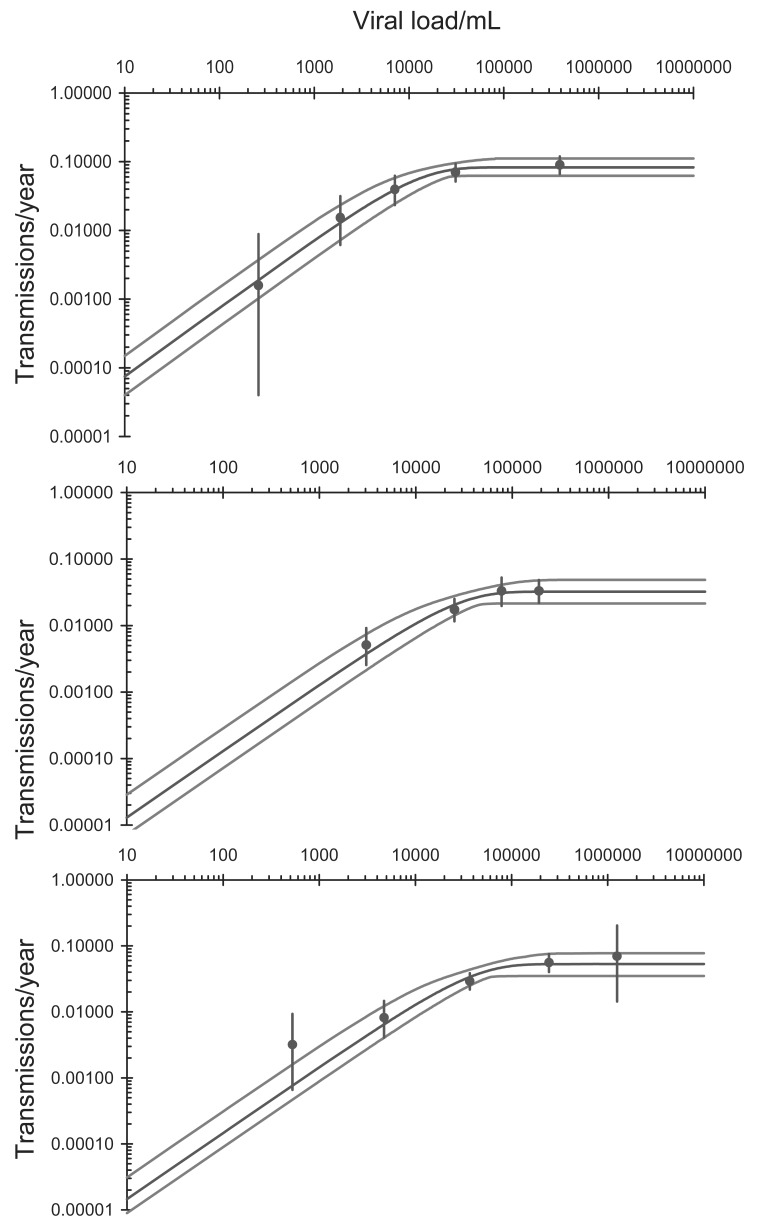
Annual probability of infection in discordant couples as a function of viral load for the three studies discussed in the text. Dots are point estimates with 95% confidence limits. Lines are maximum likelihood fits with 95% confidence bands. **A**: from a meta-analysis [106]; **B**: from HIV/HSV Transmission Study [109], **C**: Serodiscordant couples in East and Southern Africa [107]. The asymptotic transmission probabilities are **A**: 8.3% ± 1.8% per year; **B**: 3.2% ± 0.8% per year; **C**: 5.3% ± 1.3% per year. The saturation viraemiae where the extrapolated initial linear increase meets the asymptote, expressed as log_10_ copies/mL, are **A**: 4.05 ± 0.33; **B**: 4.40 ± 0.26; **C**: 4.56 ± 0.21.

Using data from a cross-sectional survey of young men in Orange Farm, South Africa, we can determine the initial distribution of viral load, and this is similar to the distributions used in a related analysis [104]. Combining this distribution with the data from the meta-analysis [106] (Fig. **7**), the overall probability that an HIV-positive person, not on ART, will infect their partner is 6.8% per year. From these data it follows that if the viral load is reduced to 400 copies/mL transmission will be reduced by 95.7%, and if viral load is reduced to 50 copies/mL transmission will be reduced by 98.9%. If we fit the data from the studies of viral load suppression in sub-Saharan Africa [101] to a cumulative log-normal distribution [108], then achieving this level of viral load suppression will reduce transmission by 89%, consistent with studies of HIV-discordant heterosexual couples in which ART reduced transmission by an estimated 92% (95% CI 43% to 100%) [109], 86% (95% CI 34% to 97%) [110] and 96% (95% CI 73% to 99%) [111].

#### Men who have Sex with Men

In a univariate analysis of MSM in the United Kingdom, highly active ART reduced transmission by 86% (95% CI 73%–93%) [112], consistent with the estimated reduction for heterosexual transmission in South Africa given above. However, a study in Australia showed that the per contact transmission probability due to unprotected anal intercourse in the era of ART was similar to estimates reported from the pre-ART era [113]. The authors did not provide information on ART coverage among HIV-infected partners, but suggest that in a population with high rates of testing and ART treatment, there could still be a substantial proportion of people with detectable viral load [114]. A review [115] highlights the lack of estimates of HIV infectiousness for MSM receiving ART and illustrates the need for more data on sexual transmission among MSM.

#### Mother-to-Child Transmission (MTCT)

ART can reduce MTCT both intra-partum and later during breastfeeding. Without ART the likelihood of intra-partum MTCT depends on the CD4 cell counts of the mother. Using data from six trials in southern, East and West Africa [35,36], the probability of MTCT increases progressively as CD4 cell counts fall from 42% (±11%) for women with a CD4 cell count below 200/mm^3^ to 15% (±4%) for those above 500/mm^3^ [35,36], giving an average over all participants in the trial of 22%. If we assume that CD4 cell counts decline linearly from 750/mm^3^, then in a steady state the proportion of women whose children will be infected at six weeks is 28%. In the same study, the reduction in transmission for women in West Africa who were given triple ART was 79% (95% CI 63%–88%) and did not vary with CD4 cell count [35,36], so that the overall rate of transmission for women on combination therapy is estimated to be 6% ± 3%. A recent study in Botswana using ART to prevent MTCT showed high rates of viral load suppression during delivery (93%–96%) and breastfeeding (92%–95%) [116]. ART provided from pregnancy through 6 months postpartum resulted in only 1.1% (0.5–2.2) of children being infected.

MTCT also depends on the mother’s viral load, and in a study of perinatal transmission [117] an acceptable fit to the data could again be obtained using Equation 2. At small values of the viral load, transmission increases linearly with viral load but then converges exponentially to an asymptotic value of 25% (±6%) at viral loads above 100,000 copies/mL. Compared to the asymptotic values, transmission is reduced by 82% (95% CI 54% to 93%) when the viral load is 400 copies/mL and 98% (95% CI 94% to 99%) when it is 50 copies/mL.

A meta-analysis of breastfeeding studies from sub-Saharan Africa showed that, without ART, transmission probabilities to infants who were HIV-negative at 4 weeks of age increased by an average of 0.8% per month, reaching 15% at 18 months [118]. A study in Mozambique showed that HIV RNA in breast milk was significantly lower in a group of women receiving nevirapine, lamivudine and zidovudine from 28 weeks of gestation compared to untreated women [119] and concluded that ART will reduce transmission through breastfeeding. A case-control study in Kenya confirmed the increased risk of infant HIV transmission among breastfeeding mothers not on ART (OR 1.7, 95% CI 1.0–2.9) [120].

Several other factors affect MTCT [120]. Having cervical or vaginal ulcers (OR 2.7, 95% CI 1.2–5.8) and mastitis (OR 3.9, 95% CI 1.2–12.7) increase the risk of infant infection. More than 2 months postpartum, transmission increases by even greater amounts if the mother has mastitis (OR 22, 95% CI 2.3–211) or breast abscess (OR 52, 95% CI 4.7–571).

### Virological Failure

In Khayelitsha, South Africa, virological failure was conservatively defined as having two consecutive viral loads above 5000 copies/mL, but patients started ART at very low CD4 cell counts, generally below 131/mm^3^. After five years, 15% had experienced virological failure, an average failure rate of 3.2% per year, and 12% had started on second line ART [121]. A systematic review of 89 studies in Africa considered virological suppression as having HIV RNA below 400 copies/mL. On this basis, 24% failed to fully suppress the virus after 1 year of antiretroviral therapy and 33% after 2 years [101], giving an average failure rate of about 15% per year. Of those with virological failure, 65% had resistance mutations for 3TC, 5% to 20% for a nucleoside reverse transcriptase inhibitor (NRTI), 5% for tenofovir, and 52% for NNRTI, so that about 10% of those on ART will develop resistance mutations to 3TC each year and about 1% to TDF. In British Columbia, adherence rates of less than 95% increased the risk of virological failure by a factor of 1.7 (95% CI 1.4-2.0) [122] and the risk of viral rebound declined by 62% per year for each additional year with full viral load suppression [123]. It is clearly important to monitor virological failure and ensure high levels of drug compliance.

### Drug Resistance

If people did not use ART or if ART suppressed the virus completely, there would be no drug resistance. Drug resistance must, therefore, peak at some intermediate level of compliance or effectiveness. In British Columbia, those who were less than 95% compliant were at greater risk of 3TC resistance (hazard ratio [HR] 4.5, 95% CI 2.6–7.9) and NNRTI resistance (HR 7.0, 95% CI 3.4 to 14.5) as compared to those who were more than 95% compliant, reinforcing the importance of achieving high levels of adherence [124]. A study in Mozambique showed that, over 12 months, 72% of people on ART were more than 95% compliant as measured by pill counts [91]. If we apply the risk of developing resistance to NNRTI as a function of compliance from the British Columbia study [124] to the compliance data for Mozambique [91], the probability of drug resistance emerging would be 70% higher than if all patients were compliant for more than 95% of the time, but would still be quite low. If new cases of drug resistance are mainly acquired [125] rather than transmitted, this further emphasizes the importance of high levels of compliance. Fortunately, predictions that drug resistance will spread rapidly in San Francisco [125,126] have not materialized.

### Acute Phase

During the initial acute phase of HIV infection the viral load is very high, but because transmission saturates as viral load increases (Fig. **7**), the increase in transmission will be less than the increase in viral load. There is evidence [127-130] that the risk of HIV transmission may be ten times greater, per unit time, during the acute phase than it is during the chronic phase [63]. However, the acute phase is estimated to last for about one to two months [130] while the chronic phase lasts for an average of about 120 months. Empirical data from a prospective cohort of patients with acute HIV infection showed a median duration of the acute phase of 15.5 days (range 3 – 67 days) [131]. A ten-fold greater risk of transmission during an acute phase lasting for one month would increase overall transmission by a factor of (10×1+1×119)/120 or 7.5%. Furthermore, this would only happen if people had several different partners per month, so that it is likely to be an upper estimate. If we assume in the extreme case that all transmission takes place during the acute phase, so that the duration of infectiousness is 1 month, and note that the doubling time of HIV in most of Africa is one year, the value of *R*_0_ from Equation 1 would be (ln(2)+12)/12 = 1.06 and a reduction in transmission of more than 6% would lead to the elimination of HIV. However, while some studies show that it is unlikely that the epidemic is driven by infection during the acute phase [132,133], others suggest that it could make a significant contribution to transmission [134]. Nevertheless, the diagnosis and management of HIV infection during the acute phase could have important implications for the control of epidemics [135].

### Individual Benefit

In public health the rights and interests of individual patients are paramount and the benefit or harm of early treatment to individual patients must be considered. Several studies have shown that starting triple-drug therapy early gives a better prognosis for individual patients [136] and none have shown the reverse. A recent analysis of data from 18 cohort studies show that delaying combination therapy until a CD4 cell count of 250/mm^3^ to 350/mm^3^ was associated with significantly higher rates of AIDS and death than starting therapy in the range of 350 to 450 CD4 cells/mm^3^ (HR 1.28, 95% CI 1.04-1.57) [137], and in France HIV-infected adults with a CD4 cell count greater than 500 cells/mm^3^ on long-term combination antiretroviral therapy reached the same mortality rates as the general population [138] after six years. A study of HIV-positive people in the US and Canada showed that deferring the start of ART until the person’s viral load fell to less than 500/mm^3^ doubled mortality over 2 to 3 years (RR 1.94, 95% CI 1.37×2.79).

Among women attending antenatal clinics in Zimbabwe [80,81], before triple therapy was available, the mortality one year postpartum in HIV-negative women was 0.13%, while HIV-positive women with a CD4 cell count of 100/mm^3^ the one-year mortality was 12% *per annum*. However, even in HIV-positive women with a CD4 cell count of 450/mm^3^, mortality was 6 times that in HIV-negative women (Fig. **4**), suggesting that they too would have benefited substantially if ART had been available and provided to them.

With increasing evidence that starting ART at a higher CD4 count may be associated with lower mortality, better immune recovery, lower rates of mother-to-child transmission, and less drug-related toxicity [139], there are likely to be significant individual benefits to starting ART early.

## IMPACT

### Transmission

Measuring the impact of treatment on population-level sexual transmission is difficult. A study in Taiwan suggested that the rate of new infections was cut by 50% after highly active ART (HAART) was made freely available in the public health sector [110,140]. A study in San Francisco suggested that after HAART became widely available, the community viral load fell by about 40% [141] and the incidence of HIV in young men fell by about 60% [142]. After HAART became widely available in Madrid, heterosexual transmission fell by 86% (95% CI 34% to 97%) [110]. A study of MSM in the United Kingdom did not find evidence of a widespread decline in HIV incidence, but their data for sites outside of London suggest that the incidence of HIV fell by 25% per annum (95% CI 1% to 49% per annum; *p* = 0.043) between 1995 and 2001 [143]. A recent study among IDUs in British Columbia [144] showed a strong association between increasing HAART coverage, decreasing viral load, and decreasing number of new HIV diagnoses per year: for every ten-fold reduction in viral load transmission fell by 14%, so that reducing community viral load by 10^3^ to 10^4^ times would reduce transmission by 36% to 45%.

ART, provided either as prophylaxis or treatment to pregnant mothers, reduces transmission to their babies. In developed countries, where ART is often combined with Caesarean section and formula feeding, MTCT transmission rates have been reduced to below 2% [15,39]. Even in resource-limited settings, ART provided to pregnant mothers and their babies can have a significant impact. In a randomised trial in Botswana, triple ART provided to HIV-infected mothers lowered the overall rate of MTCT to 1.1% (95% CI 0.5–2.2%) [116]. Further studies from the *Breastfeeding, Antiretrovirals and Nutrition* (*BAN)* trial in Malawi, and the *Kesho Bora* trial in Burkina Faso, Kenya also show reduced vertical transmission rates with maternal ART [145].

### Drug Resistance

In developed countries powerful drugs, used in combination, are driving rates of drug resistance down. A study in the United Kingdom showed that transmitted drug resistance fell from 14% to 8% between 2001-2002 and 2004 [146]. A study in France showed that between 1996 and 2006 the prevalence of transmitted drug resistance was stable over time and the authors suggest that this might be due to the increasing proportion of patients with good virologic suppression [147]. The strongest evidence that ART drugs can reduce levels of drug resistance comes from a study in British Columbia [148]. The proportion of HIV-positive people with plasma viral load below 50 copies/mL increased linearly from 65% in 2000 to 87% in 2008, while the incidence of acquired drug resistance fell exponentially at a rate of 24% per year from 1.73 to 0.13 cases per 100 person-months of therapy between 1997 and 2008, as shown in Fig. (**8**).

## CONCLUSIONS

The epidemiology of HIV, the levels of viral load suppression that can be achieved with the best available antiretroviral drugs, and the relationship between plasma viral load and transmission of HIV suggest that, with effective and early ART, HIV transmission between sexual partners or from mothers to their children can be greatly reduced. Dynamical models support the view that early ART, in combination with other methods of control, including male circumcision, vaginal microbicides, behaviour change interventions, counselling and support, should make it possible to reduce the incidence of HIV to low levels within ten years and the prevalence of HIV to low levels within forty years.

When antiretroviral drugs were first developed, therapy was delayed because of side effects, a limited understanding of drug toxicities, and concern over drug resistance. However, antiretroviral therapy has improved dramatically over the last 10 to 15 years and people on ART can now expect to live out a normal life. Therapy is now more stable, side effects are fewer, adherence is better, and resistance does not increase with earlier initiation of therapy, so that many arguments for delaying treatment are no longer valid. The weight of evidence is that individual prognosis is better with early initiation of ART, that ART reduces transmission, both sexual and mother-to-child, and that none of the potential countervailing effects, including risk compensation, poor adherence, virologic failure, residual transmission, or drug resistance are likely to overwhelm the effect of a well run and well monitored ART programme.

The studies reviewed here suggest that high levels of compliance with ART can be achieved and sustained, especially in Africa which has the greatest burden of HIV in the world. With state-of-the-art drugs, reductions in plasma viraemia of 10^4^ to 10^5^ times can be achieved and sustained. Studies of sexual transmission, mother-to-child transmission, and transmission through the exchange of blood products show that it is possible to reduce the risk of infection by about 90% or more with ART. With high levels of adherence and viral load suppression, rates of drug failure and viral rebound can be kept low and second-line drugs are available for treating those that fail first-line therapy. Even where drug resistance reached high levels as a result of the use of mono and then dual therapy in the early days of drug treatment, potent triple therapy can drive high rates of drug resistance into decline and limit the spread of resistance where it is not yet a problem.

The United Nations target for Universal Access was to ensure that 80% or more of those in need of ART would be receiving it by 2010 [26]. WHO currently recommends giving ART to all HIV-positive people with a CD4 cell count below 350/mm^3^, and to all HIV-positive women who are pregnant or breastfeeding, infants, children under the age of 2 years, and TB patients, irrespective of CD4 cell count [42,149]. At the end of 2009, just over 5 million people were receiving ART in low- and middle-income countries corresponding to 36% coverage [26]. To reach Universal Access, UNAIDS is calling for a new approach, *Treatment 2.0* [150,151], that includes the development of better combination treatment regimens, cheaper and simplified diagnostic tools, and a low-cost, community-led approach to delivery.

To achieve Universal Access, the most efficient way of finding people who are eligible for treatment will be to ensure that certain key groups are tested and fully covered: pregnant women, children born to HIV-positive mothers, TB patients and people who present with symptoms of AIDS-related diseases. Those that are HIV-positive should be counselled and offered testing and treatment. Their sexual partners should also be encouraged to receive counselling and testing.

Ensuring rapid progress towards Universal Access under present guidelines, even by 2015, will be expensive, difficult to implement, and hard to maintain and the problems should not be underestimated. It will be essential to put in place rigorous systems to monitor progress, evaluate impact and give early warning of problems that may arise. However, if this is done, the results presented here suggest that new HIV infections will be reduced by about one half in the next ten years (Fig. **2**).

The immediate need is to conduct community-level feasibility studies of using earlier treatment for prevention in which acceptability, compliance, adherence, viral load suppression, viral rebound, drug resistance, residual transmission and cost are all carefully monitored [44]. Because of the costs of running community-level trials, the scale on which they need to be done, and the importance of dealing with issues of stigma and discrimination, it will be essential to have strong and committed community involvement and control. Without the support of community leaders, prevention experts, and activists, such trials are unlikely to succeed.

Several projects on the effect of treatment on prevention in Africa are in various stages of planning and are discussed in more detail elsewhere in this issue. The *Mochudi* project in Botswana [152] will combine community-wide HIV prevention, including male circumcision, with a test-and-treat strategy. HIV-positive people with a CD4 cell count above 250/mm^3^ will be offered three-drug ART if their viral load is greater than 50,000 copies/mL, while those with a CD4 cell count below 250/mm^3^ will be referred for ART through the public health system. HIV incidence and molecular methods to elucidate transmission pathways will be used to evaluate the impact of the trial which will last for five years.

In the *iTLC (International Testing and Linkage to Care)* study, the HIV Prevention Trials Network and Family Health International are planning a 3-arm, multi-site community randomization trial [152] comparing a) the local standard of care; b) enhanced HIV testing and linkage to care with ART initiation based on the local standard of care; and c) enhanced testing and linkage to care with ART provided to all HIV-infected patients who have plasma HIV-1 RNA greater than 50,000 copies/mL.

The *TLC* (Test and Link to Care) study, also co-ordinated by the HIV Prevention Trials Network, will determine the feasibility of a community-based, enhanced test and link-to-care strategy in Washington, DC and The Bronx, New York [153]. This study will focus on expanded HIV testing, linkage-to-care, viral load suppression, prevention for positives and patient and provider surveys.

The *PopART* (Population Effects of Anti-Retroviral Therapy) trial is being developed by scientists based in the United Kingdom and in Africa to see if the widespread use of ART for all adults testing HIV positive could substantially reduce HIV transmission [154].

*TTEA* (Test and Treat to End AIDS) is a feasibility study of annual testing and immediate treatment in the Western Cape Province of South Africa being planned by scientists in South Africa, the United States and Canada [152,155].

*ANRS *(Agence Nationale de Recherche sur le SIDA et les hepatites virales) *12249-TasP *with the University of Bordeaux, the Hôpitaux Universitaires de Genève, and the Africa Centre for Health and Population Studies in South Africa is planning a pilot study of treatment as prevention which, if successful, will be developed into a community randomized controlled trial [156].

The result of the trials discussed above, as well as others being planned, will provide vital information on the potential for early treatment to stop HIV transmission, as well as the difficulties to be overcome if it is to have the greatest individual benefit and population level impact. As ART coverage continues to expand and as treatment is started earlier, people infected with HIV will experience better health and will be much less likely to infect their sexual partners. In San Francisco treatment is recommended for all HIV-positive people, irrespective of CD4 cell counts [157] and it seems likely that ART will continue to be started earlier and earlier in all countries.

With sufficient financial support, political will from national governments, support from international agencies, the commitment of the scientific community, and most importantly the active and engaged involvement of the communities of people infected with and affected by HIV and AIDS, it should be possible to stop the epidemic. ART should be at the heart of a well-constructed programme of combination prevention focussing on those interventions of proven efficacy [158].

## Figures and Tables

**Fig. (1):**
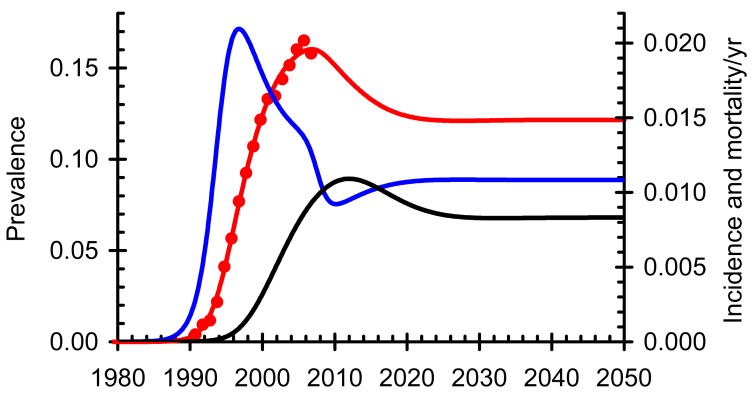
The epidemic of HIV in adults in South Africa. HIV prevalence (red), incidence (blue), mortality (black), data (red dots).

**Fig. (5):**
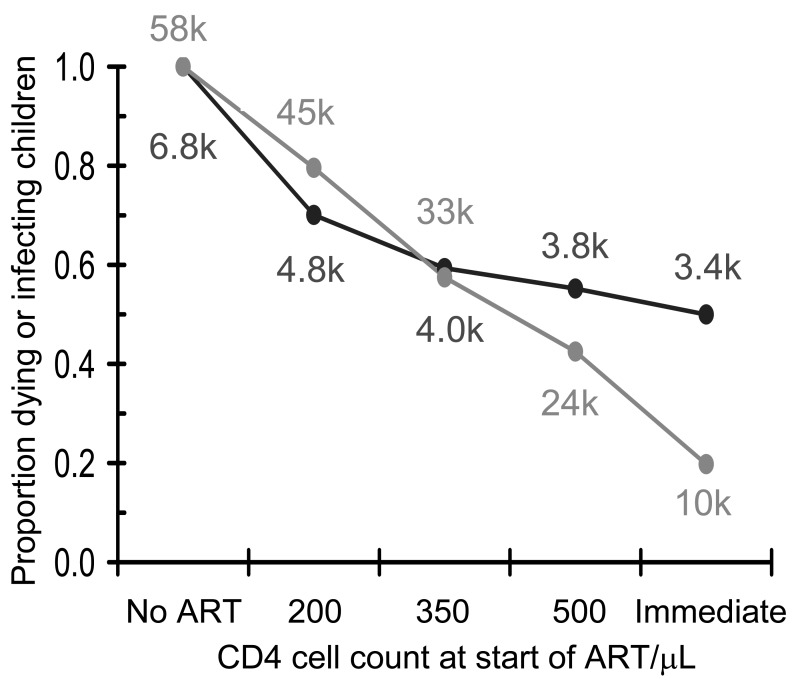
Reduction in maternal deaths after one year (black) and of infected children at 6 weeks (gray) in South Africa. For illustrative purposes the data are scaled to 1 without antiretroviral therapy (ART); the inset numbers give the annual rates. Starting treatment immediately reduces one-year mortality in mothers from 6.8 thousand to 3.4 thousand and the number of children infected at 6 weeks from 58 thousand to 10 thousand.

**Fig. (6):**
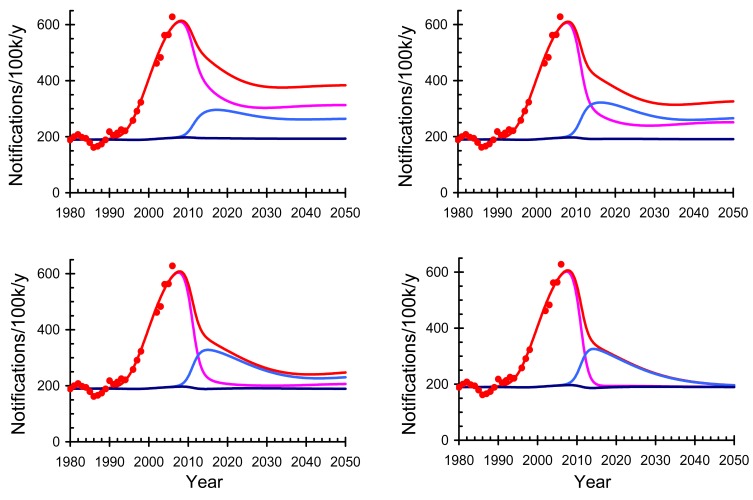
Impact of antiretroviral therapy (ART) on tuberculosis (TB) in South Africa. People start ART when their CD4 cell count is below 200 (top left), 350 (top right), 500 cells/mm^3^ (bottom left) or if they start immediately, irrespective of CD4 cell count (bottom right). TB incidence from HIV-negative people (dark blue); including HIV-positive people not on ART (pink) or HIV-positive people on ART (light blue); total (red); data (red dots).

**Fig. (8):**
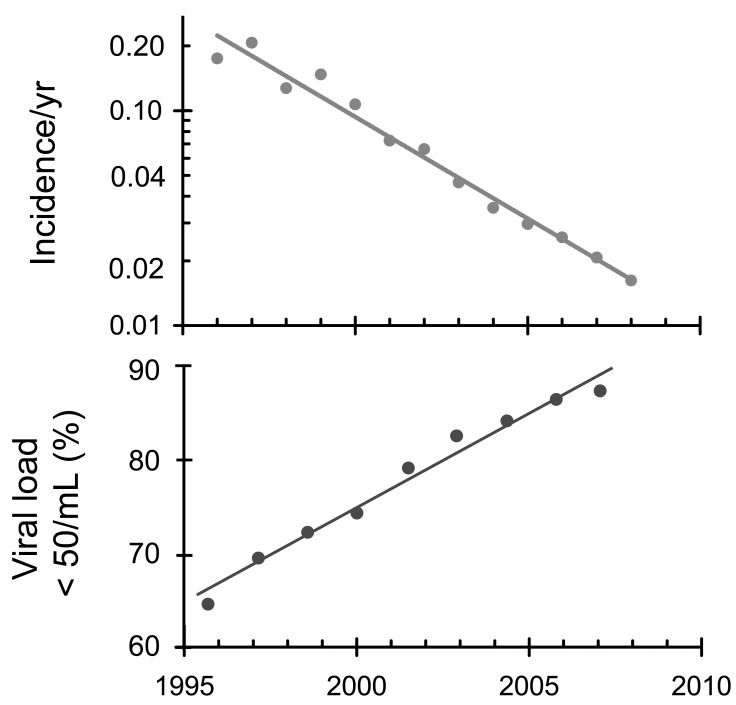
Trends in acquired drug resistance (top) and plasma viral load suppression (bottom) in British Columbia [148]. The proportion of HIV-positive people with good viral load suppression increased linearly between 1995 and 2007 (lower line) while the incidence of drug resistance fell exponentially from about 20% in 1995 to 2% in 2007 (upper line).

## ABBREVIATIONS

AIDS: = Acquired Immune Deficiency Syndrome
ANRS: = Agence Nationale de Recherche sur le SIDA et les hepatites virales (National Agency for Research on AIDS and Viral Hepatitis, France)
ART: = Antiretroviral Therapy
BAN: = Breastfeeding, Antiretrovirals and Nutrition
CAPRISA: = Centre for the AIDS Programme of Research in South Africa
CI: = Confidence Interval
DC: = District of Columbia
HAART: = Highly Active Antiretroviral Therapy
HIV: = Human Immunodeficiency Virus
HR: = Hazard Ratio
IAS: = International AIDS Society
IDU: = Injection Drug User(s)
iPrEx: = Pre-exposure Prophylaxis Initiative
IQR: = Interquartile Range
iTLC: = International Testing and Linkage to Care
MSM: = Men Who Have Sex with Men
MTCT: = Mother-to-child Transmission
NNRTI: = Non-nucleoside Reverse Transcriptase Inhibitor
OR: = Odds Ratio
PopART: = Populations Effects of Anti-Retroviral Therapy
PrEP: = Pre-exposure Prophylaxis
SACEMA: = South African Centre for Epidemiological Modelling and Analysis
TasP: = Treatment as Prevention
TB: = Tuberculosis
TDF: = Tenofovir Disoproxil Fumarate
TLC: = Test and Link to Care
TTEA: = Test and Treat to End AIDS
UNAIDS: = Joint United Nations Programme on HIV/AIDS
US(A): = United States (of America)
WHO: = World Health Organization
